# Formulation of Carbopol^®^/Poly(2-ethyl-2-oxazoline)s Mucoadhesive Tablets for Buccal Delivery of Hydrocortisone

**DOI:** 10.3390/polym10020175

**Published:** 2018-02-11

**Authors:** Leire Ruiz-Rubio, María Luz Alonso, Leyre Pérez-Álvarez, Rosa Maria Alonso, Jose Luis Vilas, Vitaliy V. Khutoryanskiy

**Affiliations:** 1 Macromolecular Chemistry Group (LABQUIMAC), Department of Physical Chemistry, Faculty of Science and Technology, University of the Basque Country, UPV/EHU, Barrio Sarriena, s/n, 48940 Leioa, Spain; leyre.perez@ehu.eus (L.P.-Á.); joseluis.vilas@ehu.es (J.L.V.); 2 Analytical Chemistry Department, Faculty of Science and Technology, University of the Basque Country, UPV/EHU, Barrio Sarriena, s/n, 48940 Leioa, Spain; marialuz.alonso@ehu.eus (M.L.A.); rosamaria.alonso@ehu.eus (R.M.A.); 3 School of Pharmacy, University of Reading, Whiteknights, P.O. Box 224, Reading RG6 6AD, UK

**Keywords:** poly(2-ethyl-2-oxazoline), Carbopol^®^, mucoadhesion, interpolymer complexes

## Abstract

Poly(2-ethyl-2-oxazoline) has become an excellent alternative to the use of poly(ethylene glycol) in pharmaceutical formulations due to its valuable physicochemical and biological properties. This work presents a formulation of poorly-water soluble drug, hydrocortisone, using interpolymer complexes and physical blends of poly(2-ethyl-2-oxazoline)s and two Carbopols^®^ (Carbopol 974 and Carbopol 971) for oromucosal administration. The swelling, hydrocortisone release and mucoadhesive properties of a series of tablet formulations obtained by combination of different Carbopols with poly(2-ethyl-2-oxazoline)s of different molecular weights have been evaluated in vitro.

## 1. Introduction

The development of dosage forms containing poorly-water soluble drugs remains a challenging task for formulation science. Several approaches have been reported to improve the solubility and transport of lipophilic drugs, including particle size reduction, preparation of solid dispersions, microemulsions or complexation with various pharmaceutical excipients [1,2,3]. Polyethylene glycols (PEGs) have been widely used to formulate poorly-water soluble drugs [4] and in other pharmaceutical applications [5]. However, lately some unfavorable effects related to PEG use have emerged, such as adverse side effects in the body, or unexpected changes in the pharmacokinetics [6]. In addition, some cases of hypersensitivity and laxative effect when using PEG in oral dosage forms have been reported [6,7]. Consequently, various synthetic and natural hydrophilic polymers have been considered as potential alternatives to PEG. In the last years, poly(2-oxazoline)s (POZs) have been highlighted as promising polymers and studies have been focused on the use of these polymers for biomedical applications [8,9,10]. The main advantages of POZs include: they are easy to synthesize; they do not form peroxides; and they are stable at room temperature and in water. They could be easily cleared from the body and are not excessively hydroscopic [11,12,13]. On the contrary, PEG can be accumulated in some organs and form vacuoles due to its desiccant nature [14,15]. In addition, poly(2-oxazoline)s are highly versatile materials with a capability to form functional materials and structures depending on the nature of the pending side chain used. These various structures have received increased interest in the use of these polymers in the last years [10,12,13].

Oral delivery of drugs remains the most popular route for medicine administration. Some aspects such as control and ease of administration by the patient, rapid removal in case of toxic effects, limited local enzymatic activity, low irritation problems, high versatility for local or systemic release systems increased the interest in these dosage forms [16,17]. One of the more effective strategies is to deliver drugs in the oral cavity through the adhesion of dosage forms to the buccal mucosa. These dosage forms could be used for the treatment of some conditions directly in the oral cavity and also could be used for systemic drug delivery [18,19]. Among the various dosage forms explored for buccal administration, tablets are still the most commonly used, because of several advantages they offer. Mucoadhesive polymers have a crucial role in tablet formulations due to their ability to adhere to specific regions of the mucosal surface and prolonged residence time of the formulation. Carbomers, usually named Carbopols, are weakly cross-linked polymers of acrylic acid with excellent mucoadhesive properties, being extensively used in a dosage forms design. The presence of high amounts of carboxylic groups in their structure allows water absorption and their high swelling degree. These carboxylic groups are dissociated in a basic environment (p*K*_a_ around 6), and cause chain repulsion, polymer swelling and formation of gels. 

However, it is often difficult to control the drug release from Carbopol^®^ based matrixes and their pH sensitivity could complicate the correlation between the in vitro and in vivo drug release [20,21]. Several approaches have been reported to use hydrogen-bonded interpolymer complexes based on Carbopols^®^ and non-ionic polymers to develop dosage forms capable of overcoming these disadvantages [22]. 

The aim of this work is to develop a formulation for poorly-water soluble drug, hydrocortisone, based on poly(2-ethyl-2-oxazoline)s and Carbopols^®^ for buccal delivery. The lack of mucoadhesion of poly(2-ethyl-2-oxazoline)s was improved by forming interpolymer complexes with Carbopols that present excellent adhesion to oral mucosa. In this work, we evaluated swelling, hydrocortisone release and mucoadhesive properties of a series of tablet formulations based on different Carbopols^®^ and poly(2-ethyl-2-oxazoline)s of different molecular weights in vitro.

## 2. Materials and Methods

### 2.1. Reagents and Solutions

Poly(2-ethyl-2-oxazoline)s (50, 200, and 500 kDa; named as 50, 200 and 500 in the text, respectively) and hydrocortisone (HC) were purchased from Sigma-Aldrich Irvine, UK) and Carbopol 974 (highly cross-linked, 28,400–39,400 cP) and Carbopol 971 (moderately cross-linked, 4000–11,000 cP) were obtained from Lubrizol (Hazelwood, UK). HPLC grade acetonitrile (ACN) was purchased from Teknokroma (Barcelona, Spain). Sodium chloride, calcium chloride, potassium chloride, sodium hydrogen carbonate and potassium dihydrogen phosphate were obtained from Merck (Darmstadt, Germany) and used for preparation of artificial saliva fluids. A Milli-Q water purification system from Millipore (Bedford, MA, USA) was used. The drug hydrocortisone and internal standard dexamethasone (DXM) were purchased from Sigma-Aldrich (St Louis, MO, USA). Standard stock solutions of drug and internal standard were prepared in acetonitrile at a concentration of 1000 mg/L. Working solutions were prepared by dilution from stock solutions with the mix of excipients and polymers used in the pharmaceutical formulation of tablets (magnesium state, poly(2-ethyl-2-oxazoline) and Carbopols 974 and Carbopol 971) in artificial saliva.

### 2.2. Complex Formation

Interpolymer complexes were prepared by mixing separate 0.1 wt % polymer solutions in deionized water. Solutions were mixed to give different unit molar ratios of the polymer components. The complexation between Carbopol and POZ was evaluated in water without adjusting pH and also at pH 2 (which was adjusted by addition of 0.1 mol/L HCl). The obtained interpolymer complexes were left for 2 days in the media, and then they were separated, washed twice with the solvent and freeze-dried in a Heto PowerDry LL3000 Freeze Dryer (Thermo Scientific, Loughborough, UK) for at least two days. The composition with the maximum concentration yield was selected for the tablet formulation. 

Infrared spectra of the interpolymer complexes and pure components were recorded using Nicolet Nexus FTIR spectrophotometer Thermo Scientific, Loughborough, UK) in KBr pellets, at a resolution of 4 cm^−1^ and 32 scans. Glass transition temperatures (*T*_g_) were determined using Mettler-Toledo Differential Scanning Calorimeter, DSC 822^e^ (Gießen, Germany), heating from 20 to 180 °C at 20 °C·min^−1^, and the *T*_g_ was taken as the mid-point of the curve inflection.

### 2.3. Tablet Formulation and Preparation

Three different tablet formulations were prepared in this study: (1) physical mixtures of pure polymers, Carbopols and poly(2-ethyl-2-oxazoline)s at 50/50 *w*/*w*; (2) interpolymer complexes of Carbopols/Poly(2-ethyl-2-oxazoline)s at 50/50 *w*/*w* prepared in water; and (3) interpolymer complexes of Carbopols/POZ at 50/50 *w*/*w* prepared at pH 2. Polymer mixture or interpolymer complex powders were mixed with hydrocortisone (5 *w/w* %) and magnesium stearate (2.5 *w/w* % as a lubricant) in a Willy A Bachofen AG Maschinenfabrik mixer (Muttenz, Switzerland). The powders were compressed into tablets using a Riva Minipress (Hampshire, UK), single punch tablet press (Riva, Hampshire, UK) filled manually and press settings selected to preserve similar tablet strength between batches. An average weight of the tablets was 50 ± 1 mg and the average diameter of tablets was 6 ± 0.3 mm. 

### 2.4. Mucoadhesion of the Tablets

The mucoadhesive properties of the tablets were analyzed using a TA.XT Plus Texture Analyser (Stable Micro System, Surrey, UK). Freshly isolated porcine buccal mucosal tissues taken from female Great White pigs were obtained from local abattoir. Before each test, the tissue was equilibrated at 37 ± 0.5 °C in a solution simulating saliva (0.43 g NaCl, 0.22 g CaCl_2_, 0.75 g KCl, 0.20 g NaHCO_3_ and 0.9 g KH_2_PO_4_ dissolved in 1 L of deionized water, pH 6.75) [23]. The tablets were manually attached to the texture analyzer probe by using double-sided adhesive tape. The porcine tissue was set on the mucoadhesion rig and moisturized with artificial saliva. The tablets were put in contact with the mucosa for 1 min with a downward force of 0.1 N. Then, the probe was withdrawn from the tissue at a 1 mm·s^−1^. Each experiment was repeated at least three times, the detachment force and the total work of adhesion, calculated from the area under the detachment curve, were measured. Mucoadhesion results were analyzed using one way, non-paired ANOVA (analysis of variance) with Tukey test.

### 2.5. Swelling Behavior

The swelling properties of the tablets were studied in a simulated saliva. The test was performed using metallic mesh baskets (2 cm diameter, 3 cm height) placed inside glass vials (3.5 cm in diameter and 6 cm in height). The tablets were weighed and placed into the metallic baskets. The initial weight of the samples and the weight of each basket used (after being immersed into the medium were used for the test and wiped always in the same manner) were accurately recorded. The tablets were kept in excess of medium for 3 days and weighed regularly, by weighing the baskets with the tablet samples inside. The weights of the baskets were taken into consideration to calculate the weight of the swollen samples. The swelling ratio (*SR*) of the tablets was calculated using the following equation (Equation (1)) [24]:

(1) $$SR=\frac{W_{s}-W_{i}}{W_{i}}$$

where *W*_i_ is the initial tablet weight and *W*_s_ is the weight of the sample after additional swelling, respectively.

### 2.6. Hydrocortisone Release from the Tablets

#### 2.6.1. Instruments and Chromatographic Conditions

For the analysis of hydrocortisone released from the tablets, 2690 high performance liquid chromatography equipment (HPLC) and a 484 diode array detector (DAD) (Waters, Milford, MA, USA) were used. A Sartorius CP224S Scale (Goettegen, Germany) was used with a precision of ±0.0001. The pH measurements were performed on a GLP22 Crison pH meter (Barcelona, Spain). Dissolution tests were carried out at 37 °C in a USP dissolution apparatus II (paddle) using a Sotax AT 7smart Dissolution Tester (Nordring, Switzerland) in accordance with the US and European Pharmacopeia with paddle method.

Chromatographic separation was achieved on a C-18 (50 mm × 4.6 mm, 3.5 µm) Waters XBridge column (Waters Corporation, Milford, MA, USA), using an isocratic mode with a 77:23 (H_2_O:acetonitrile) mobile phase at a flow rate of 0.6 mL/min. A sample aliquot of 10 μL was injected into the column at 30 °C and the working wavelength was 245 nm [24,25,26,27].

#### 2.6.2. Dissolution Tests

An in vitro drug release study from tablets, to simulate the physiological conditions at the buccal mucosa level, was carried out. The dissolution profiles of the tablet samples were obtained in 500 mL of simulated saliva (pH 6.75). The paddle rotational speed was set at 100 rpm at a constant temperature bath of 37.0 ± 0.5 °C. The dissolution experiment was initiated by placing the sample in the dissolution vessel. Sample aliquots of 5 mL were withdrawn at specific intervals (0, 1, 3, 5, 24, 48, and 72 h) replaced with an equal volume of fresh medium. The aliquots were filtered through a 0.45 µm PTFE filter prior to the injection in the HPLC system (Method validation in the Supplementary Materials, Figure S1) [24,28].

## 3. Results and Discussion

### 3.1. Fabrication of Poly(2-ethyl-2-oxazoline)/Carbopol Interpolymer Complexes

**Figure 1:**
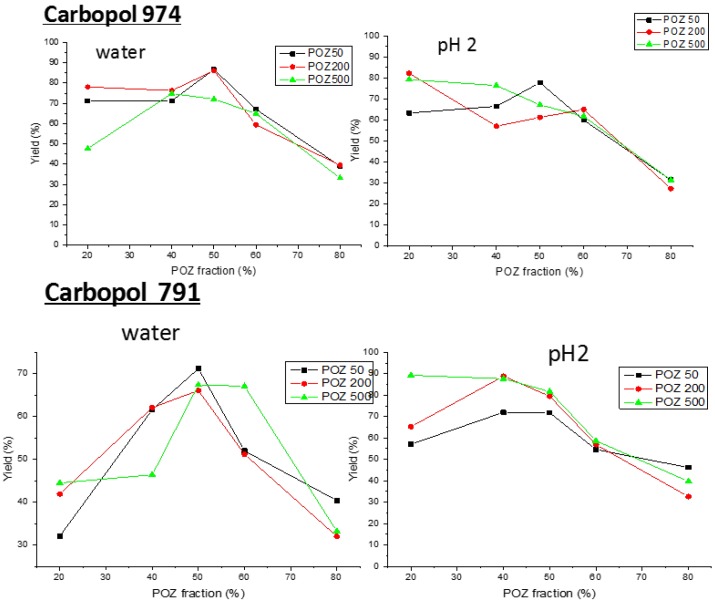
Yields of interpolymer complexes (wt %) for different Carbopol/poly(2-ethyl-2-oxazoline) systems in water and at pH 2.

The cooperative interaction between poly(2-ethyl-2-oxazoline)s and Carbopols leads to the formation of a gel like precipitate, being a white powder when it is dried. This precipitate is an interpolymer complex formed due to hydrogen bonding between carboxylic groups of Carbopol and amide groups of poly(2-oxazoline). The yield of different interpolymer complexes formed for each combination of the 974 and 971 Carbopols with POZ of different molecular weights have been studied both in water (final pH 5) and at pH 2 (Figure 1). Since Carbopol 974 is a more cross-linked sample presenting a higher viscosity than Carbopol 971 (weakly cross-linked), variations on the swelling and release properties could be expected. In addition, poly(2-ethyl-2-oxazoline) samples used in this study have three molecular weights (50, 200, and 500 kDa) that could also affect the yield of complexation. However, the maximum yield for all evaluated systems was obtained around 50/50 *w*/*w* feed composition independent of the molecular weights, so this feed composition was selected for production of interpolymer complexes used for tablet formulation.

**Figure 2:**
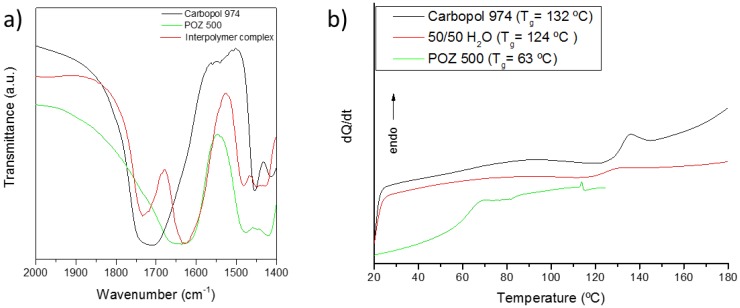
Analysis of Carbopol 974/poly(2-ethyl-2-oxazoline) 500 complex, and pure components by: (**a**) FTIR; and (**b**) DSC.

The formation of interpolymer complexes between the complementary polymers was confirmed by FTIR spectra and by differential scanning calorimetry (DSC) (Figure 2). The FTIR bands of the carbonyl region are shown, where Carbopol present a carboxyl stretching band corresponding to its self-association is located at 1709 cm^−1^ and a stretching band of amide I of POZ at 1643 cm^−1^. In the interpolymer complex, a shift of the C=O bands could be observed to 1732 cm^−1^, while the amide I band shifts to 1625 cm^−1^. These bands are related to hydrogen bond formation between the carboxyl groups of Carbopol and the amide groups of poly(2-ethyl-2-oxazoline) [29,30,31,32]. Figure 2b shows the DSC thermograms of Carbopol 974, POZ 500 and their interpolymer complex formed in water. Carbopol 974 presents a *T*_g_ at 132 °C, whereas the *T*_g_ of poly(2-ethyl-2-oxazoline) 500 is located at 63 °C. The interpolymer complexes of these polymers formed in water present an intermediate glass transition of 124 °C, similar to the changes observed in other interpolymer complexes formed via hydrogen bonding [33,34,35].

### 3.2. Mucoadhesion Studies

**Figure 3:**
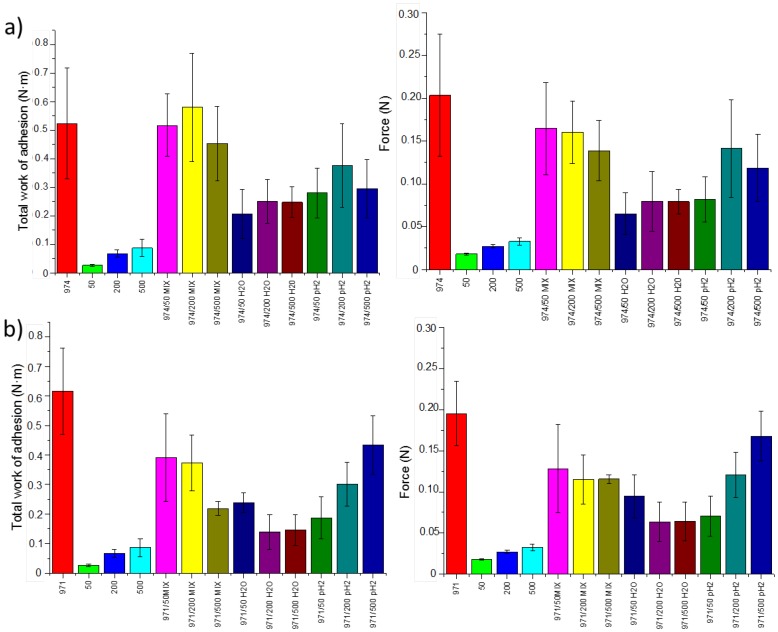
The total work of adhesion (**left**) and detachment force (**right**) on porcine buccal tissues at 37 °C for: (**a**) Carbopol 974/poly(2-ethyl-2-oxazoline) tablets; and (**b**) Carbopol 971/poly(2-ethyl-oxazoline) tablets.

The mucoadhesion is an important property in the development of drug delivery systems for buccal, nasal or ocular administration. Several hydrophilic polymers containing functional groups capable to form hydrogen bonds, such as carboxylic acids, have good adhesion properties [36,37,38,39]. Different methods can be used to evaluate the adhesion of polymers to a mucosal tissue. One of the most common methods used to analyze the mucoadhesion of solid dosage forms is based on the measurement of the so-called detachment force. The force of detachment and the total work of adhesion are used by many researchers to evaluate the mucoadhesive properties of solid dosage forms [36]. In the present work, the maximum detachment force and the total work of adhesion for Carbopol/poly(2-ethyl-oxazoline) tablets to mucosal tissue were determined (Figure 3). The data generated in these experiments indicated that there is a linear correlation between the maximal detachment force and the total work of adhesion in almost all of the samples. 

Overall, pure poly(2-ethyl-2-oxazoline)s exhibit poor mucoadhesive properties, with their total work of adhesion being between 0.018 and 0.032 N·m, increasing slightly with increase in the polymer molecular weight. Some increase in the adhesiveness of poly(2-ethyl-2-oxazoline)s with larger molecular weight could be related to improved ability of larger macromolecules to entangle with mucin biomacromolecules. On the contrary, Carbopols exhibit excellent mucoadhesive properties due to the ability of their carboxylic groups to form strong hydrogen bonds with the oligosaccharide chains present in the mucin [40,41]. This physical interaction promotes strong adhesion of the tablets to mucosal tissue; the values of the force of detachment for tablets prepared from pure Carbopol 974 and Carbopol 971 are 0.20 ± 0.04 and 0.18 ± 0.04 N, respectively. The total work of adhesion values for Carbopol 974 and Carbopol 971 are also quite high: 0.52 ± 0.19 and 0.62 ± 0.15 N·m, respectively. 

Physical mixtures of Carbopols and poly(2-ethyl-2-oxazoline)s (50/50 *w/w*) were prepared and their mucoadhesive properties were evaluated. The values of the detachment forces and the total work of adhesion of physical mixtures are greater than those recorded for pure POZ tablets. However, these mixtures are heterogeneous, since there is no interaction between the components, and the tablet erosion (analyzed in the following section) was greater than in the rest of the formulations. Usually, interpolymer complexes present intermediate properties of polymer components, as it has been shown for the *T*_g_. Since interpolymer complexes present enhanced stability and resistance to erosion due to polymer-polymer interactions, an improvement in POZ mucoadhesion and stability by the complexation with Carbopols could be expected. Figure 3 shows an improvement in mucoadhesive properties of POZs after complexation with Carbopols regardless of their molecular weight. With respect to the pH of the medium during complexation process, it is noteworthy that when the main excipient of the tablet is the interpolymer complexes obtained in water, they present inferior mucoadhesion than that of the physical mixture. However, when the interpolymer complexes are obtained in acidic media (pH fix to 2), the adhesion of the tablets increases, being similar to the ones obtained for physical mixtures. This fact indicates a diminished mucoadhesion for complexes prepared in the swollen state of Carbopol polymer at neutral pH due to stronger interaction between complementary polymers. Thus, interpolymer complexation leads to materials with high mucoadhesive properties similar to those of physical mixtures but homogeneous and presenting slower erosion.

In the systems based on Carbopol 974, the dosage forms prepared with physical mixtures and interpolymer complexes at pH 2 were not significantly different from pure Carbopol 974 (*p* > 0.05) independently of POZ molecular weight. However, the tablets based on Carbopol 971 complexes with POZ were significantly different from pure Carbopol 971 (*p* < 0.05), except for the interpolymer complexes formed by high molecular weight POZ 500 at pH 2, which present a mucoadhesion similar to that of the physical mixtures (*p* > 0.05). In this case, the molecular weight of poly(2-ethyl-2-oxazoline)s complexed with Carbopol 971 seem to linearly increase their mucoadhesion at pH 2. The lower cross-linking of the Carbopol varies the influence of the molecular weight of POZ on the ionization of the network and the access to carboxylic groups capable to interact with the mucin. Similar phenomenon was also observed in the swelling studies.

### 3.3. Swelling Studies

**Figure 4:**
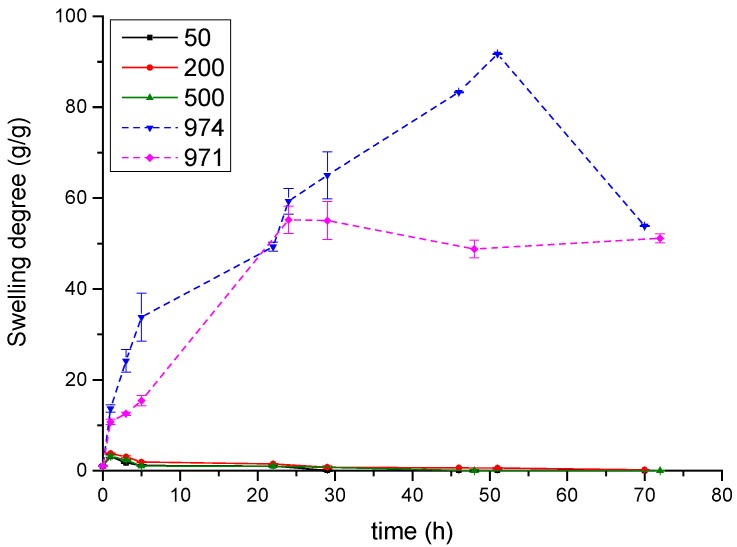
Swelling degree of pure polymers in simulated saliva.

**Figure 5:**
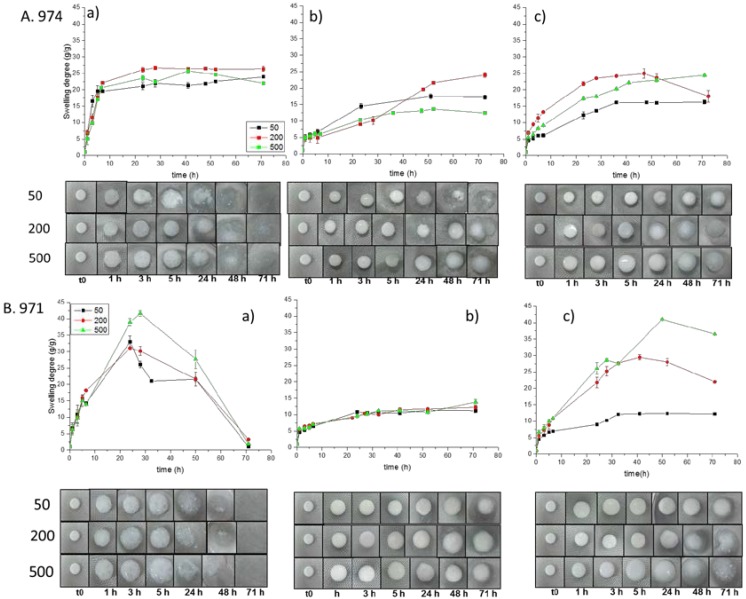
Swelling degree and photographs of the tablets of Carbopols/Poly(2-ethyl-2-oxazoline)s in saliva at 37 °C at regular time intervals: (**a**) physical mixture; (**b**) interpolymer complexes formed in water; and (**c**) interpolymer complexes formed at pH 2 (A Carbopol 974; and B. Carbopol 971).

The swelling behavior of the formulated tablets is a crucial factor since it directly affects the polymer solubility and the drug release process when tablets are located in the mouth. The polymer swelling improves the consolidation of the tablets on the mucosal tissue increasing the mobility of the molecules and facilitating the penetration and release of the drug in the mucus layer. The swelling behavior of the tablets in an ion-containing solution (simulating the saliva) was monitored for three days. Figure 4 shows the variation of the swelling degree of Carbopols and poly(2-ethyl-2-oxazoline)s. The difference between the components could be observed; Carbopols present greater swelling capability, whereas poly(2-ethyl-2-oxazoline) presents poorer swelling, <5 g/g. Figure 5 shows the swelling degree and the photographs of the erosion of the tablets of the prepared Carbopols/poly(2-ethyl-2-oxazoline)s complexes taken at regular time intervals. Physical mixtures showed erosion during the first 3 h of swelling, while interpolymer complexes remain stable until 24–48 h and no erosion was observed for complexes of Carbopol 971 formed at neutral pH during the first 71 h (Figure 5). These results show greater erosion of the tablets with weaker interpolymer interactions, and suggest enhanced Carbopol/POZ interaction leads to stable tablets, for the mentioned sample, as will be corroborated and explained below.

The cross-linking density of Carbopols seems to have more influence on the swelling capability than the presence of poly(2-ethyl-2-oxazoline). Comparison of Figure 5A,B shows that tablets based on Carbopol 971 exhibit greater swelling degree than 974, being more prominent for tablet physical mixtures and interpolymer complexes formed at pH 2. This is in accordance with the more crosslinked structure of Carbopol 974 in comparison with Carbopol 971. Surprisingly, Carbopol 971 complexed with POZs under neutral pH conditions displayed a limited swelling for all the cases. This behavior can be ascribed to the H-bonding with POZs, which is more prominent when complexation is carried out with open and swollen Carbopol networks leading to a better interaction with POZs, that is the case for moderately cross-linked Carbopol 971 at neutral pH, when ionization of their carboxylic groups promotes maximum swelling of pure polyacid network.

As can be observed in Figure 5A, for more crosslinked Carbopol 974, physical mixtures and interpolymer complexes regardless of the solution pH used during the complexation, displayed a similar swelling uptake around 20%, what it takes to conclude that Carbopol structure limits swelling regardless the presence of POZ. Thus, it seems that the greater cross-linking of used polyacid network, which restricts Carbopol swelling, limits Carbopol/POZ interaction and consequently their influence on their complexes swelling. However, the effect of those restricted H-bonding could be observed on a retardation effect in those tablets formed by interpolymer complexes, which was more pronounced for complexes formed in water at pH 5, corroborating the above described role of Carbopol swelling in H-bonding interaction with POZs.

Analyzing the swelling of tablets containing POZ samples of different molecular weights, a different effect according to employed Carbopol structure and pH of the medium could be observed. In this sense, it could be observed that tablets with restricted swelling, because of stronger Carbopol/POZ interactions, did not show any dependence of their swelling on POZ molecular weight. Certainly, these limiting interactions result from an open network state during complexation, which facilitates POZ diffusion into Carbopol network. Thus, the behavior of tablets based on interpolymer complexes of Carbopol 971 in water should be particularly noted, which corresponds to the sample with the most swollen Carbopol prior to the complexation. All of them present very similar swelling degree, less than 10%, independent of POZ molecular weights and lower than the other cases, because POZ macromolecules penetration within polyacid network is favored for all the studied molecular weights (Figure 5B(b)). However, when Carbopol 971 network is collapsed as a consequence of a lower ionization degree at pH = 2, only POZ macromolecules with the lowest molecular weight can get inside Carbopol network promoting swelling restricting interactions (Figure 5B(c)). Similar effect was observed for Carbopol 974/POZs tablets, in which molecular weight of POZ did not show any clear effect on the swelling of the tablets when complexes were prepared in water (Figure 5B(b)). That is, in the swollen state of Carbopol, but limited swelling was found for the lowest molecular weight when complexes were formed at pH = 2 (Figure 5B(c)).

### 3.4. Dissolution Test

**Figure 6:**
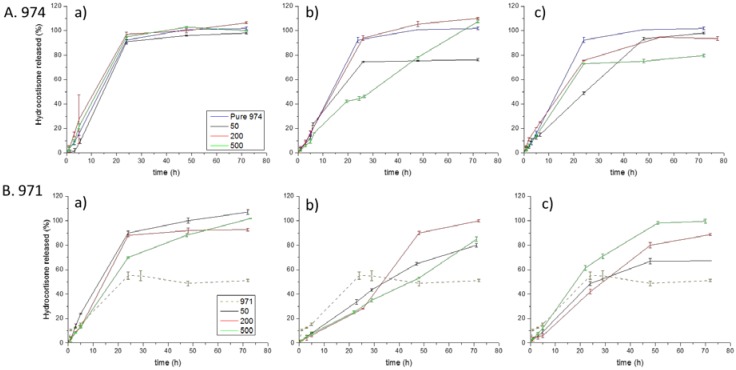
Release profiles of hydrocortisone from the tablets composed of poly(2-ethyl-2-oxazoline)s/Carbopols in simulated saliva at 37 °C: (**a**) physical mixture; (**b**) interpolymer complexes formed in water; and (**c**) interpolymer complexes formed at pH 2. (A Carbopol 974; and B. Carbopol 971).

The release of hydrocortisone from the tablets was evaluated under sink conditions at pH 6.75. Figure 6 shows the dissolution profiles from the tablets based on Carbopol 974 and Carbopol 971 as well as their complexes and physical mixtures with POZ (enlarged release profiles of the first 10 h are shown in Figure S2). A direct correlation between the swelling of interpolymer complexes samples and HC release was observed. Hydrocortisone released faster from the tablets composed of a physical mixture of the polymer components than when interpolymer complexes were used. However, this is even more pronounced for the complexes formed by Carbopol 971, which can be explained according to their swelling behavior resulting from an enhanced complexation with POZ. Besides, it can be observed that there is no variation in the release profile from pure Carbopol 974 to physical mixtures of Carbopol 974 and POZs. These results are related to the wettability and the swelling degree of the tablets, since similar results were obtained in the swelling studies, and indicate that the main driving effect for this behavior is related to Carbopol 974. When using Carbopol 974 interpolymer complexes, the release rate slightly decreased compared to the physical mixtures and to pure Carbopol 974. However, highest decrease was measured for complexes that displayed lowest swelling. As was expected, Carbopol 971 complexes, for which Carbopol/POZs interaction were favored, showed lower swelling, HC release was slower and total HC released content was lower.

The nature of interpolymer complexes, seems not only to change the swelling degree, but also varies the release profile of HC from the tablets. By analyzing Figure 6, a linear drug release could be observed in the beginning of the swelling process.

Hydrocortisone release from the tablets based on interpolymer complexes formed in water using weaker cross-linked Carbopol 971 displays a linear character in almost all the studied systems with slight variations between the different molecular weight of POZ. Similar observations were reported by Park et al. [21] in their study of theophylline release from chitosan/Carbopol 971, where at pH 6.8 the molecular weight of chitosan did not show any influence on the drug release, resulting in linear release profiles. These linear release profiles have been ascribed in previous studies to poorly-water soluble nature of the drugs used (similar to hydrocortisone), which tend to partition into less polar material (POZ in our case) [20,42].

## 4. Conclusions

In this work, the tablet formulations for buccal delivery of a poorly-water soluble drug, hydrocortisone, were developed based on poly(2-ethyl-oxazoline)s and Carbopols. The mucoadhesive properties of poly(2-ethyl-oxazoline)s have improved by their complexation/mixing with Carbopols. Swelling, erosion and hydrocortisone release for different tablet formulations using interpolymer complexes as main excipient have been evaluated. The tables formed from interpolymer complexes obtained in water present better swelling and release properties. The study suggests that Carbopol/poly(2-ethyl-oxazoline) tablets could present an adequate swelling and hydrocortisone release to be used in applications in which a prolonged release is required such as periodontitis disease.

## Supplementary Materials

The following are available online at http://www.mdpi.com/2073-4360/10/2/175/s1, Figure S1: Chromatogram of HC and DXM solutions at a concentration of 1 mg/L of HC and DXM, Figure S2: Release profiles, first 10 hours, of hydrocortisone from the tablets composed of poly(2-ethyl-2-oxazoline)s/ Carbopols in simulated saliva at 37 °C: (a) physical mixture, (b) interpolymer complexes in water and (c) interpolymer complexes at pH 2.
