# Supplementary material for: Formulation of Carbopol®/Poly(2-ethyl-2-oxazoline)s Mucoadhesive Tablets for Buccal Delivery of Hydrocortisone
Source: Polymers (Basel). 2018 Feb 11;10(2):175. doi: 10.3390/polym10020175 (PMC6415335; doi:10.3390/polym10020175)
Supplement: Supplementary file 1 [file polymers-10-00175-s001.pdf]

Supplementary Materials

Formulation of Carbopol®/poly(2-ethyl-2-oxazoline)s mucoadhesive tablets for buccal delivery of hydrocortisone

Leire Ruiz-Rubio[1]*, María Luz Alonso[2], Leyre Pérez-Álvarez[1], Rosa María Alonso[2], José Luis Vilas-Vilela[1], Vitaliy V. Khutoryanskiy[3]*

[1] Macromolecular Chemistry Group (LABQUIMAC), Department of Physical Chemistry, Faculty of Science and Technology, University of the Basque Country, UPV/EHU, Barrio Sarriena, s/n 48940 Leioa, Spain; leyre.perez@ehu.eus (L.P.-Á.); joseluis.vilas@ehu.es (J.L.V.)

[2] Analytical Chemistry Department, Faculty of Science and Technology, University of the Basque Country, UPV/EHU, Barrio Sarriena, s/n, 48940 Leioa, Spain; marialuz.alonso@ehu.eus (M.L.A.); rosamaria.alonso@ehu.eus (R.M.A.)

[3] School of Pharmacy, University of Reading, Whiteknights, P.O. Box 224, Reading RG6 6AD, UK

* Correspondence: leire.ruiz@ehu.eus (L.R.-R.); v.khutoryanskiy@reading.ac.uk (V.V.K.); Tel.: +34-946017972 (L.R.-R.); +44-1183786119 (V.V.K.)

Received: 25 December 2017; Accepted: 7 February 2018; Published: 11 February 2018

* Correspondence: leire.ruiz@ehu.eus ; Tel.: +34-946017972; v.khutoryanskiy@reading.ac.uk, Tel.: +44-1183786119

HPLC-DAD Method Validation for Pharmaceutical Analysis

The validation of the HPLC-DAD method developed was performed following the European Medicines Agency (EMA) guidelines for bioanalytical method validation.[1] The analytical method was validated in terms of selectivity, carry over, LLOQ (limit of quantification), linearity, accuracy, precision and stability

The selectivity of the method was evaluated in the first place. The developed method was tested to determine hydrocortisone without interference of other components in the chromatogram. To see if the excipients have any absorption maxima at 245 nm, the excipients were injected in the chromatographic system and the obtained chromatograms were compared with the ones corresponding to HC and DXM. The signal obtained in the blank matrices must be lower than 20% of the response of HC at the LLOQ and 5% of the response of DXM.

Linear concentration range was calculated from the calibration curve built with 6 calibration solutions ranging from LLOQ and 2 mg/L and with a constant DXM concentration of 1 mg/L. Data corrected peak area (HC peak area/DXM peak area)-concentration were treated by means of linear regression method. The calculation of the limits of detection (LOD) was performed using the formula ($y_{blank} + 3\sigma_{blank}$), while for LLOQ the formula ($y_{blank} + 10\sigma_{blank}$) was used, where y_{blank} corresponds to the average of the areas obtained for the blank solution and σ_{blank} to the deviation of 10 replicates of blank solution.

During validation carry-over was assessed by injecting blank samples after calibration standard at the upper limit of quantification. The response in the blank sample was then compared with the response at the LLOQ, and was considered acceptable if the signal obtained at the HC retention time was under 20% of the signal at the LLOQ and 5% for the internal standard.

The experiment repeatability and accuracy was assessed within a single run, in a day, and in different runs, between days. It was determined by analysing 6 samples for 3 concentration levels, which are covering the calibration curve range: LLOQ, 1 and 2 mg/L, respectively. Accuracy was expressed as relative error (%RE=(average value -real value)*100/real value), and precision were evaluated as relative standard deviation (%RSD=standard deviation*100/average). The acceptance criterion for accuracy and precision was <15% from the nominal concentration and 20% for LLOQ standard, respectively.

Evaluation of stability of HC and DXM stock solutions was performed for a period of 7 months. The mean concentration obtained should be within ±15% of the nominal concentration.

Method Validation

In the optimum chromatographic conditions, HC eluted at 4.2 min while the internal standard DXM at 9.1 min, as can be seen in Figure S1.

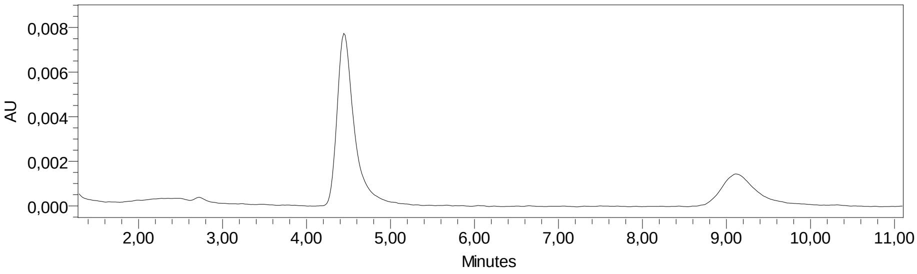

Figure S1. Chromatogram of HC and DXM solutions at a concentration of 1 mg/L of HC and DXM.

Chromatographic conditions are collected in section 2.6.

The values of selectivity, carry over, linearity, accuracy, precision and stability meet the established criteria by European Medicines Agency (EMA) guideline. Analytical method results to be selective, free of carry over, with a repeatability and an accuracy in terms of %RSD and RME lower than 15% for HC and 5% for DXM. LOD and LLOQ were 78 and 81 ng/mL, respectively. HC and DXM stock solutions were stable during 7 months.

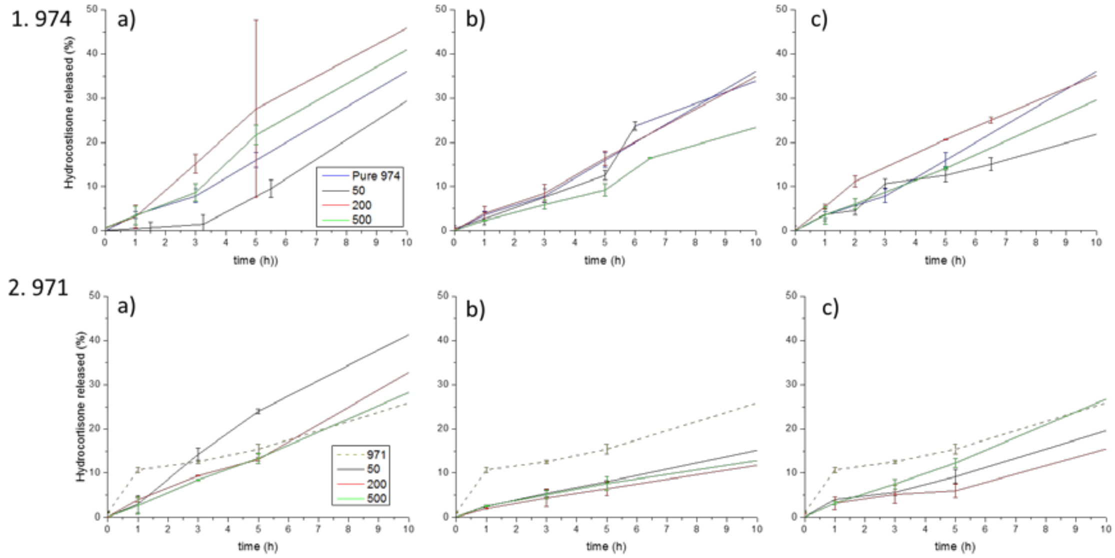

Figure S2. Release profiles, first 10 hours, of hydrocortisone from the tablets composed of poly(2-ethyl-2-oxazoline)s/ Carbopols in simulated saliva at 37 ºC: a)physical mixture, b) interpolymer complexes in water and c) interpolymer complexes at pH 2.

References

[1] Committee for medical products for human use, in: Guideline on Bioanalytical Method Validation, European Medicines Agency, London, UK, 2011.
